# Therapeutic Dilemmas Faced When Managing a Life-Threatening Presentation of a Myocardial Bridge

**DOI:** 10.1155/2022/8148241

**Published:** 2022-04-11

**Authors:** Debbie Falconer, Sariha Yousfani, Anna S. Herrey, Pier Lambiase, Gabriella Captur

**Affiliations:** ^1^Royal Free Hospital, Pond Street, London NW3 2QG, UK; ^2^Barts Heart Centre, W Smithfield, London EC1A 7BE, UK; ^3^MRC Unit for Lifelong Health and Ageing at UCL, 1-19 Torrington Place, Fitzrovia, London, UK

## Abstract

*Background*. Myocardial bridges are congenital abnormalities, where a segment of coronary artery travels intramyocardially, rather than the typical epicardial course. The overlying muscle segment is termed “the bridge”. Most myocardial bridges are asymptomatic, but some can result in myocardial ischaemia, arrhythmias, and sudden cardiac death. *Case Presentation*. A 31-year-old male with no past medical history presented to our tertiary cardiac centre following an out-of-hospital ventricular fibrillation arrest. Coronary angiography and computed tomography of the coronary arteries revealed a 2 cm myocardial bridge overlying the left anterior descending (LAD) artery. An exercise echocardiogram demonstrated severe apical ballooning and hypokinesis during peak exercise, with corresponding ST-segment elevation, resolving on rest. Options for medical therapy of a symptomatic myocardial bridge include beta blockers, calcium channel blockers, ivabradine, or a combination thereof. Surgical interventions include deroofing the bridge and revascularisation of the affected region with bypass grafting. However, a lack of trial data comparing medical regimens and surgical interventions makes it difficult to ascertain the most effective management strategy for each patient. There was disagreement between experts at different tertiary centres over the optimal management of this patient. He was treated with multiple regimes of medical therapy with ongoing ischaemia on stress testing, before undergoing a negative stress test on amlodipine, diltiazem, and isosorbide mononitrate. It was felt that no further intervention was necessary at this time given his exercise test was now negative for ischaemia. However, after seeking a second opinion, he underwent surgical intervention with bypass grafting of his left anterior descending artery, followed by implantation of an implantable cardiac defibrillator. Subsequently, an angiogram postsurgery demonstrated concomitant spasm of the LAD and he was resumed on medical therapy with calcium channel blockers and nitrates. *Discussion*. Without randomised trials, it is impossible to determine the optimal management strategy for each patient. It is possible that some patients with myocardial bridges are not being trialled on optimal medical therapy prior to undergoing invasive and irreversible interventions.

## 1. Introduction

Myocardial bridges are congenital abnormalities where a segment of coronary artery travels intramyocardially, rather than the typical epicardial course. The overlying segment of muscle is termed the “myocardial bridge”. The commonest vessel involved is the left anterior descending (LAD) artery [1]. Estimated prevalence varies widely between studies, but one review of 1,056 patients at autopsy found bridges in 26% of subjects [2]. The vast majority of myocardial bridges are asymptomatic, but case reports indicate that bridges may clinically present as myocardial ischaemia [3], arrhythmia [4], and sudden cardiac death [5, 6].

Such complications arise from one or more of the following pathophysiological mechanisms:

1. Systolic myocardial contraction which directly compresses the underlying artery thus limiting perfusion distally. This obstruction has been shown to extend from systole into diastole, where the majority of coronary artery perfusion usually occurs [7]. In tachycardic states such as exercise where diastole is shortened, there will be a further reduction in the perfusion time for the bridged segment of coronary artery, thereby worsening symptoms. Other coexisting factors such as diastolic dysfunction or coronary artery disease can exacerbate the perfusion-requirement mismatch [8]
2. Abnormal haemodynamics within the “bridged” vessel, and reduced coronary flow reserve in the vessel distal to the bridge which can aggravate the supply-demand mismatch and result in myocardial ischaemia [9]
3. Reversal of systolic flow through the bridged artery which leads to high shear wall stress and accelerated atherosclerosis in the segment proximal to the bridge [10]

Management of symptomatic myocardial bridges can be challenging, as there are currently no guidelines on the optimal therapeutic strategy.

## 2. Case Report

A 31-year-old male presented to the emergency department following an out-of-hospital ventricular fibrillation (VF) arrest, which occurred whilst running. He had no past medical history of note and no family history of heart disease or sudden death. He recalled feeling light-headed immediately before collapsing but denied chest pain or palpitations. He reported no previous episodes of loss of consciousness or dizziness with exercise.

**Figure 1:**
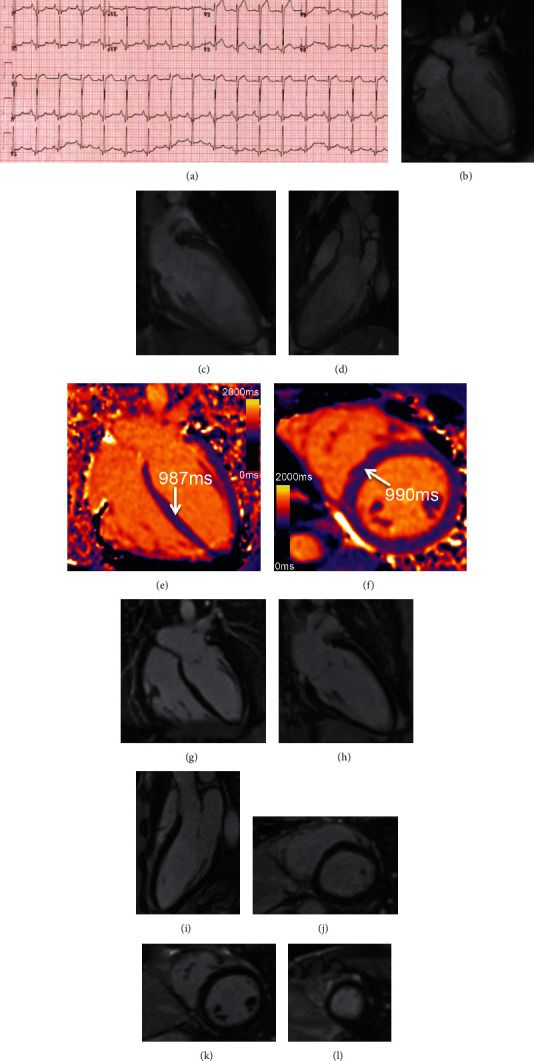
(a) resting ECG on admission showing early repolarization in V1-V3. (b–l) Nonperfusion cardiovascular magnetic resonance. (b–d) Cine steady-state free precession images. (e, f) T1 mapping by MOLLI 5s (3s) 3s showing normal native myocardial T1 at 1.5 T (normal reference range at our center 950-1060 ms). (g–l) Late gadolinium enhancement images with motion correction showing no focal myocardial fibrosis.

He was rescued by a bystander's cardiopulmonary resuscitation with minimal delay, and 1 shock was administered when paramedics arrived on the scene. This was followed by return of spontaneous circulation with a normal Glasgow coma scale. His electrocardiogram (ECG; Figure 1(a)) demonstrated sinus rhythm and early repolarization in V1-V3 and his troponin peaked at 292 *μ*g/ml. On examination, his heart sounds were normal with no added sounds. There was no evidence of heart failure clinically.

**Figure 2:**
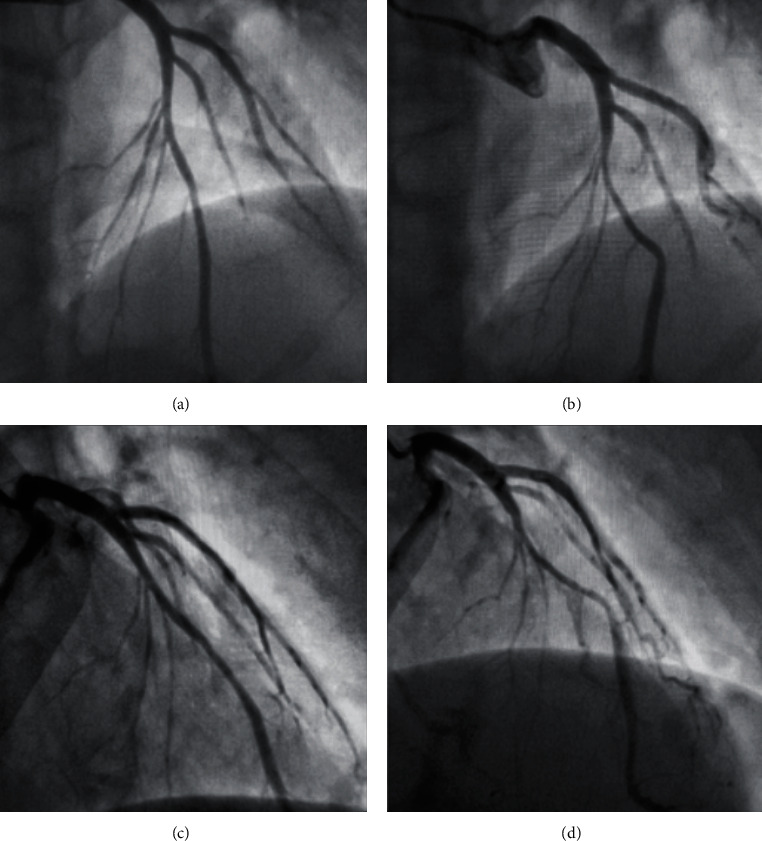
Angiographic images taken on admission to our tertiary centre. Panels (a) and (c) demonstrate images of the left anterior descending artery (LAD) in diastole. Panels (b) and (d) demonstrate the same views during systole with compression of the mid-LAD.

He was taken directly to the catheterization laboratory of our tertiary hospital, where a resting bedside echocardiogram demonstrated no regional wall motion abnormalities and normal left ventricular wall thickness. Angiography demonstrated a muscle bridge overlying a 2 cm portion of the LAD artery [Figure 2]. There was mild atherosclerotic disease of the LAD, felt unlikely to be the cause of the arrest, and a normal appearance of the other vessels. A nonstress cardiovascular magnetic resonance scan showed normal myocardial tissue characteristics and no focal fibrosis [Figures 1(b)–1(l)]. Computed tomography coronary angiography demonstrated 2 cm of the LAD running intramyocardially, with an overlying bridge. He was started on bisoprolol 2.5 mg, aspirin 75 mg, and atorvastatin 40 mg.

**Figure 3:**
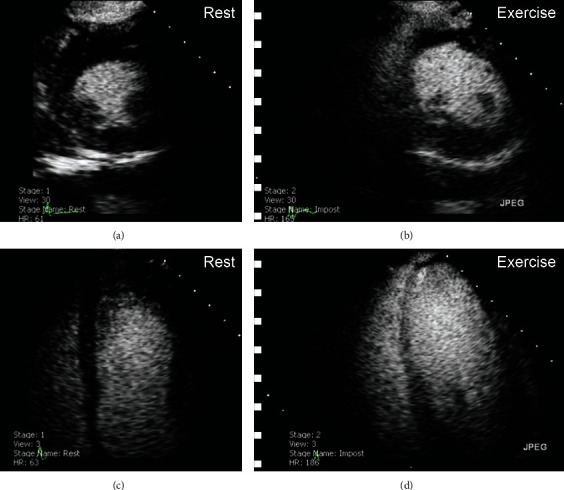
Stress echocardiogram with contrast. Parasternal mid left ventricular short axis slices (a, b) and 4-chamber views (c, d). Images (a) and (c) are taken at rest. Images (b) and (d) are taken at peak exercise, demonstrating apical ballooning.

A stress echocardiogram was performed (Bruce full protocol). At peak exercise, there was ST elevation seen in V3-6, with corresponding severe apical hypokinesia of the left ventricle [Figures 3(a)–3(d)] that resolved at rest. Following discussion at the joint cardiology and cardiothoracic multidisciplinary team meeting (MDT), amlodipine 5 mg and isosorbide mononitrate 30 mg were added. On this medical therapy, his stress echocardiogram was repeated but the inducible ischaemia persisted. After further MDT discussion, it was felt he should undergo single-vessel coronary artery bypass grafting but the patient declined and opted instead for continued medical therapy.

In the weeks that followed, his medical therapy was uptitrated and altered to bisoprolol 5 mg, amlodipine 20 mg, and ivabradine to 5 mg BD. The exercise stress test was repeated but this still resulted in 7 mm of anterior ST elevation that resolved with intravenous nitrates.

After further MDT discussion, he was switched to a regime consisting of amlodipine 10 mg, diltiazem 360 mg, and isosorbide mononitrate 30 mg. On this therapy, he was asymptomatic and a subsequent exercise stress test was negative for ischaemia. He was discharged on this medication regimen. Following extensive MDT discussion, it was felt that an implantable cardiac defibrillator (ICD) was not indicated given the absence of ischaemia on repeat testing, and that the VF arrest had occurred in the context of a reversible cause.

The patient sought a second opinion at another tertiary centre which led to a repeat angiogram, this time with fractional flow reserve (FFR) assessment of the LAD which was positive. He thus underwent cardiac surgery, involving unroofing of the bridge and a left inferior mammary artery (LIMA) bypass graft to the LAD. Postoperatively, an angiogram with acetylcholine provocation was performed, demonstrating spasm of the left anterior descending artery, and he was restarted on amlodipine, diltiazem, and isosorbide mononitrate. A subcutaneous ICD was also implanted at this stage.

At most recent follow-up, the patient has returned to his prior level of exercise and is asymptomatic.

## 3. Discussion

Difficulties in this case arose from the lack of consensus on the management of symptomatic bridges in the context of proven ischaemia. As there are no clear guidelines, management of our patient was based on multiple conflicting opinions which differed between centres and specialists.

Pharmacological therapy in the form of beta-blockers is generally first-line due to their negative chronotropic and inotropic effects. They will reduce the arterial compression and the diastolic shortening seen in tachycardia, increasing arterial perfusion. Calcium channel blockers are often added next to reduce concomitant vasospasm [11]. Ivabradine can be used in combination with the aforementioned drugs, exerting a chrononegative effect via *I*_*f*_ [10]. Beta-blockers have been shown to reduce systolic compression and anginal symptoms in a small study of 15 patients [12], but no studies have demonstrated improvements in morbidity or mortality of bridge patients. Also, no trials have compared the efficacy of the aforementioned drugs. The sparsity of data makes it difficult to predict which medical regime will work best for each individual patient. Therefore, patients presenting with major adverse cardiac events may be offered surgical intervention prematurely and before an effective drug regimen is found.

Surgical or interventional approaches including myotomy, coronary artery bypass grafting and percutaneous coronary intervention are usually reserved for patients in whom medical management is unsuccessful [11]. Surgical myotomy involves resection of the overlying muscle fibres, whilst bypass grafting involves anastomosing the LIMA to the LAD. A small study of 31 patients who underwent either myotomy or bypass grafting demonstrated no ischaemia on postoperative exercise testing of any patient after a follow-up of 31 months, along with no early or late complications, suggesting surgical approaches are safe and effective in managing symptomatic bridges [13]. It is not reported, however, which medical therapies were utilised and deemed unsuccessful before proceeding to surgery. The benefit derived from surgery in our case is unclear, as he was forced to restart medical therapy postoperatively following the detection of coronary artery spasm on repeat angiogram. It is also possible that given his young age, he may require repeat surgery on the graft, re-exposing him to a substantial surgical and anaesthetic risk, whilst the benefits from the original surgery remain unknown.

In the management of our patient, opinions also differed regarding the need for an ICD. To date, no malignant ventricular arrhythmias outside the initial presentation have been detected although the device may well impact on the patient's quality of life in the long term.

It is generally accepted that nitrates are contraindicated and should not be used to treat ischaemia caused by myocardial bridges, as they may lead to proximal vessel dilatation and flow reversal, further compromising distal vessel perfusion [8]. A small study of 3 patients demonstrated that administration of intravenous nitrates exacerbated systolic compression of bridged segments of coronary arteries during angiography [14]. However, when used in combination with diltiazem and amlodipine, nitrates did not result in worsening of ischaemia on stress testing, or of our patient's symptoms. Indeed, on this regimen, his stress test was negative for ischaemia and he was asymptomatic. Given he was later diagnosed with vasospasm of the LAD, the nitrates may have been useful in preventing concomitant spasm of the bridged segment.

Another consideration is that patients may undergo repeated stress testing to determine the efficacy of medical therapy—a potentially dangerous and anxiety-inducing procedure. Our patient underwent 3 positive stress tests, each accompanied by ST elevation in the anterior leads thus risking a cardiac arrest before a successful drug combination was found. Clearer guidelines on optimal management would reduce the need for repeat testing, allowing treating teams to select the correct management the first time.

Another management strategy that can be overlooked in this group of patients is aggressive coronary artery disease risk factor modification. Given the increased risk of atherosclerosis in the area proximal to the bridge, antiplatelet and statin therapy should be considered as well as optimization of conventional risk factors.

This case highlights the urgent need for trials comparing the efficacy of different medical regimes and surgical interventions to determine which patient will benefit from which treatment strategy. All studies thus far are small, nonrandomised, and focus on the surgical or interventional treatment strategies. We also need to understand the safety of sole medical therapy in patients with a myocardial bridge following a major cardiac event, because the avoidance of surgery and device implantation may be beneficial, if at all possible.

## 4. Conclusion

We present the case of a patient who suffered VF arrest secondary to a myocardial bridge of the LAD. Various treatment strategies were trialled including medical therapy, surgical unroofing and bypass, and ultimately ICD implantation. Without randomised data, some patients with myocardial bridges are not being trialled on optimal medical therapy prior to undergoing invasive and irreversible interventions.
